# Specialties of the Present Day No Novelty

**Published:** 1861-02

**Authors:** 


Specialties of the Present Day no Novelty.—The system of special practice, which is becoming so prevalent at the present time, existed among the ancient Egyptians, for Herodotus speaks of their having doctors for almost every part of the body, of which the eye and other organs are particularly mentioned. Our specialism would seem then, to be merely a revival of an ancient though not enlightened practice.—*London Lancet.*

